# Skin Autofluorescence, as a Measure of AGE Accumulation in Individuals Suffering from Chronic Plaque Psoriasis

**DOI:** 10.1155/2018/4016939

**Published:** 2018-09-27

**Authors:** Karolina Kopeć-Pyciarz, Irena Makulska, Danuta Zwolińska, Łukasz Łaczmański, Wojciech Baran

**Affiliations:** ^1^Department of Dermatology, Venereology and Allergology, Wroclaw Medical University, Poland; ^2^Department of Paediatric Nephrology, Wroclaw Medical University, Poland; ^3^Institute of Immunology and Experimental Therapy, Polish Academy of Sciences, Poland

## Abstract

**Background:**

Psoriasis is currently regarded as a chronic systemic inflammatory disease associated with increased cardiovascular risk. Advanced glycation end products (AGEs) contribute to the development of atherosclerosis.

**Objectives:**

The aim of the study was the assessment of skin autofluorescence (SAF), as a measure of AGE accumulation, in individuals suffering from chronic plaque psoriasis without any comorbid conditions.

**Methods:**

A study group consisted of 70 patients with chronic plaque psoriasis without any comorbid conditions and 59 healthy controls, matched by age and gender. AGE accumulation was assessed by SAF (AGE Reader, DiagnOptics BV) which is a validated and noninvasive technique. Relations between SAF and some clinical and laboratory data were assessed.

**Results:**

SAF was positively correlated with age both in patients with psoriasis and controls (*R* = 0.722, *p* < 0.00001 and *R* = 0.613, *p* < 0.00001, respectively). There was significantly increased SAF in patients with psoriasis with elevated levels of C-reactive protein (CRP) and increased erythrocyte sedimentation rate (ESR) compared to controls (*p* < 0.00001; *p* < 0.00001, respectively, after adjustment to age). Increased SAF was found in psoriatic patients with prediabetes (HbA1c 5.7–6.4%) compared to controls (*p* < 0.0012, after adjustment to age).

**Conclusion:**

Systemic inflammation (increased CRP level), prediabetes, and aging may influence enhanced AGE accumulation in patients with psoriasis without any comorbidities. SAF may be considered as a useful, noninvasive method to identify patients with psoriasis at increased cardiovascular risk.

## 1. Introduction

Psoriasis is a chronic, relapsing immune-mediated inflammatory disease of the skin that affects approximately 2% of the population worldwide. The growing number of evidence indicates that psoriasis is a chronic systemic inflammatory disease associated with increased cardiovascular risk.

Advanced glycation end products (AGEs) are a group of heterogeneous compounds formed by endogenous nonenzymatic glycation and oxidation (glycoxidation) of proteins, lipids, and nucleic acids [1–4]. Accumulation of AGEs increases in chronic hyperglycemia and also in oxidative stress associated with chronic inflammation. Increased formation and accumulation of AGEs have been found in diabetes, chronic renal failure, and some chronic immune-mediated inflammatory diseases, such as rheumatoid arthritis [3–8]. A chronic inflammatory process may lead to the oxidative stress and formation of reactive carbonyl compounds that may be partly transformed to AGEs [1–3]. It has been shown that accumulation of AGEs is associated with accelerated atherosclerosis and vascular complications [3, 4, 6–9]. AGE accumulation in an extracellular matrix results in increased rigidity and reduced elasticity of the vascular wall [2–4]. Furthermore, interactions of AGEs with receptor for advanced glycation end products (RAGE) have been shown to be implicated in the development of endothelial dysfunction and atherosclerosis [3, 9–11]. AGEs acting through RAGE induce activation of intracellular signaling pathways leading to enhanced production of proinflammatory cytokines, adhesion molecules, vasoconstriction, and coagulation [11].

AGE accumulation can be assessed by skin autofluorescence (SAF) following the principles of AGE Reader, which is a validated and noninvasive technique. SAF measured by AGE Reader significantly correlates with specific AGEs, such as pentosidine, N-(carboxymethyl)-lysine (CML) and N-(carboxyethyl) lysine (CEL), in skin biopsies [3, 4, 12–15]. Increased SAF has been shown to be associated with vascular complications and was an independent predictor of cardiovascular morbidity and mortality in patients with diabetes, chronic renal failure, and cardiovascular diseases [4, 16–23]. Furthermore, SAF was related to arterial stiffness and the insulin level in healthy population [24, 25].

The objective of the present study was the assessment of SAF, as a measure of AGE accumulation, in individuals suffering from chronic plaque psoriasis. To evaluate the effect of psoriasis on AGE accumulation more precisely, we excluded from the study psoriatic patients with comorbid conditions associated with enhanced AGE accumulation, such as diabetes, dyslipidemia, chronic renal failure, cardiovascular diseases, and smoking habit. Furthermore, we assessed relations between SAF and the severity of skin involvement, level of C-reactive protein (CRP), erythrocyte sedimentation rate (ESR), body mass index (BMI), age, gender, levels of LDL, and levels of glycated hemoglobin (HbA1c).

## 2. Materials and Methods

### 2.1. Study Group

The study group consisted of 70 patients with chronic plaque psoriasis and 59 healthy controls, matched by age and gender. Subjects underwent clinical and demographic evaluations at the time of recruitment (Table 1). All patients and controls were Caucasians. Patients did not receive any systemic therapy or phototherapy at least 8 weeks prior to the initiation of the study. Severity of disease was assessed by using Psoriasis Area and Severity Index (PASI) and body surface area (BSA). The patients did not have psoriatic lesions on the inner surface of at least one forearm. 49 patients had type I psoriasis (25 women and 24 men) and 21 type II psoriasis (10 women and 11 men). From each subject, approximately 15 ml of blood was taken for laboratory tests (complete blood count, lipid profile, fasting glucose, glycated hemoglobin (HbA1c), C-reactive protein (CRP), and erythrocyte sedimentation rate (ESR)). Exclusion criteria included cardiovascular disease (defined as history of coronary artery disease, myocardial infarction, stroke, and chronic heart failure), chronic renal or liver disease, diabetes mellitus, malignancies, dyslipidemia, smoking habit, and uncontrolled hypertension. Subjects who reported smoking at least one cigarette per day during the last year were classified as smokers. Dyslipidemia was defined as an elevated level of triglyceride and total or low-density lipoprotein (LDL) cholesterol. Body mass index (BMI) was calculated as the ratio of weight (kg) to height (m) squared (kg/m^2^). According to WHO criteria, subjects with BMI ≥ 30 were considered as obese. The study was approved by the local bioethical committee according to Helsinki Declaration.

### 2.2. Skin Autofluorescence

Noninvasive measurement of skin autofluorescence (SAF) was assessed by AGE Reader, DiagnOptics BV (Groningen, the Netherlands), according to the guidelines [12]. Approximately 4 cm^2^ of the skin of the ventral site of the lower arm, protected from surrounding light, in room temperature was illuminated with a wavelength of 300–420 nm. Measurements were performed at uninvolved skin to prevent influences by the presence of skin lesions. Emission light and reflected excitation light were measured with a spectrometer (AVS-USB2000; Avantes, Eerbeek, the Netherlands). The autofluorescence was calculated by dividing the mean emitted intensity per nm by the mean reflected excitation intensity and expressed in arbitrary units (AU). The SAF value is calculated by automated analysis. Results are mean values of three measurements performed for each patients [12]. Laboratory tests were performed using standard assays.

### 2.3. Statistical Analysis

The results were statistically analyzed using the computer program Statistica 13.0 (Windows 10) and GraphPad Prism version 5.0. The mean, median, maximal, minimal, and standard deviations of values were calculated. Student's *t*-test (for normally distributed data) or Mann-Whitney *U* test (for not normally distributed data) were used to analyze continuous variables. Data was adjusted to age. The significance of differences between categorical variables was determined by the Pearson Chi-square test, Chi-square with Yates correction test, or Fisher's exact test. The relations between continuous variables of interest were assessed by Spearman's rank correlation coefficient. Statistical significance was set at *p* < 0.05.

## 3. Results

**Figure 1:**
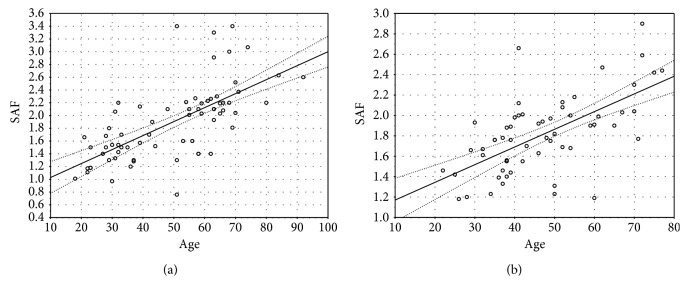
(a) Significant positive correlations between SAF and age in patients with psoriasis (*R* = 0.722, *p* < 0.00001). (b) Significant positive correlations between SAF and age in controls (*R* = 0.613, *p* < 0.00001).

**Figure 2:**
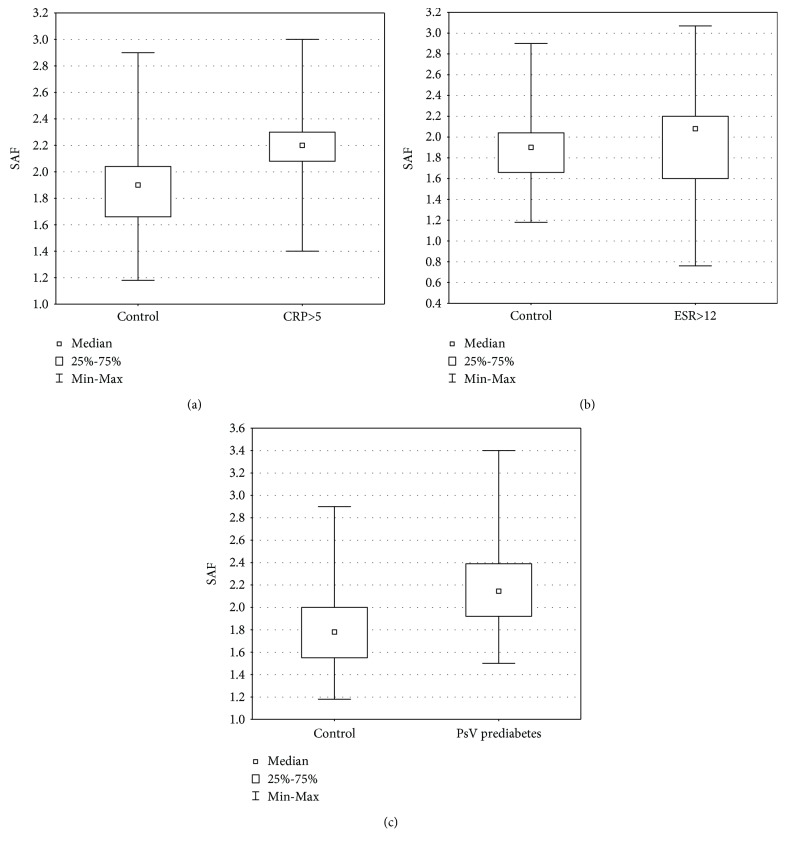
(a) Increased SAF in patients with psoriasis with elevated CRP levels (>5 mg/dl) compared to controls (*p* < 0.00001, *Z* = −19.120). (b) Increased SAF in patients with psoriasis with increased ESR (>12 mm) compared to controls (*p* < 0.00001, *Z* = −12.504). (c) Increased SAF in psoriatic patients with prediabetes (HbA1c from 5.7% to 6.4%) compared to controls (*p* = 0.0012, *Z* = −3.26). Data adjusted to the age.

Detailed characteristics of patients with psoriasis are shown in Table 2. We found significant positive correlations between SAF and age both in patients with psoriasis and controls (*R* = 0.722, *p* < 0.00001; *R* = 0.613, *p* < 0.00001, respectively) (Figure 1). There was no statistically significant difference in SAF values between the whole group of patients with psoriasis and controls (1.887 ± 0.6 vs. 1.816 ± 0.38). The mean ± SD value of CRP in patients with psoriasis was 5.96 ± 9.79 mg/dl, and 19 patients had elevated levels of CRP (CRP >5 mg/dl, according to the local laboratory). There was significantly increased SAF in psoriatic patients with elevated levels of CRP compared to controls (2.037 ± 0.45 vs. 1.816 ± 0.38; *p* = 0.039), and this difference was even more significant after adjustment to age (*p* < 0.00001, *Z* = −19.12) (Figure 2(a)). The mean ± SD value of ESR in patients with psoriasis was 16.64 ± 13.90, and 34 patients had increased ESR (ESR > 12 mm, according to the local laboratory). There was significantly increased SAF in psoriatic patients with increased ESR (*n* = 34) compared to controls (2.059 ± 0.52 vs. 1.816 ± 0.38; *p* = 0.012), and this difference was even more significant after adjustment to age (*p* < 0.00001, *Z* = −12.5) (Figure 2(b)). There were no significant differences in SAF because of gender, both in patients with psoriasis and controls (Table 3). We did not find any relevant correlations between SAF and severity of skin involvement assessed with PASI and BSA. There was a significant relationship between SAF and BMI in patients with psoriasis (*R* = 0.297, *p* = 0.0129). We did not find any relationship between SAF and BMI in controls. The mean ± SD value of HbA1c in patients with psoriasis was 5.381 ± 0.47%. Prediabetes was defined as the concentration of HbA1c from 5.7% to 6.4% (39–46 mmol/mol). Psoriatic patients with prediabetes (*n* = 22) had significantly increased SAF compared to controls (2.213 ± 0.53 vs. 1.816 ± 0.38; *p* = 0.0006), and this significant difference remains after adjustment to age (*p* = 0.0012, *Z* = −3.238) (Figure 2(c)).

## 4. Discussion

Although psoriasis is generally euglycemic disease, a chronic immune-mediated inflammatory process may lead to the increased AGE accumulation in individuals with psoriasis [26–28]. Increased AGE accumulation has been found in metabolic abnormalities, such as dyslipidemia and diabetes [16–18]. Numerous studies showed increased prevalence of dyslipidemia and diabetes in individuals suffering from psoriasis [29, 30]. However, to investigate the impact of psoriasis on AGE accumulation more precisely, we excluded from the study psoriatic patients with comorbidities, such as diabetes, dyslipidemia, and obesity. We found positive correlations between SAF and age, both in psoriatic patients and healthy controls. Correlations between SAF and age were previously reported [31, 32]. Although we did not find any significant difference in SAF between the whole group of patients with psoriasis and controls, we showed that individuals suffering from psoriasis, with elevated CRP levels and increased ESR, had significantly increased SAF as compared to controls. It may suggest the impact of the systemic inflammation on AGE generation and accumulation in individuals with psoriasis. Correlations between SAF and CRP have been previously reported [33]. Furthermore, several studies showed positive correlations between CRP levels and severity of skin involvement in psoriasis [34]. However, in the present study, we did not find such relationship. In the present study, we showed the positive correlation between SAF and BMI in patients with psoriasis. We did not find such a correlation in controls. In previous study, den Engelsen et al. [31] demonstrated higher SAF in nondiabetic obese individuals as compared to controls (nonobese subjects). Fatty tissue, as a source of a wide range of proinflammatory mediators (e.g., IL-6), may contribute to development of systemic inflammation and impact the AGE accumulation in patients with psoriasis. According to the concept of psoriatic march, systemic inflammation in patients with psoriasis subsequently contributes to the insulin resistance, endothelial dysfunction, and increased cardiovascular risk [27]. Increased AGE accumulation may constitute the link between systemic inflammation and increased cardiovascular risk in patients with psoriasis. Furthermore, we analyzed the level of HbA1c in nondiabetic individuals with psoriasis. We showed that individuals with prediabetes had significantly increased SAF as compared to controls.

To the best of our knowledge, this is the first study conducted in Caucasian psoriatic patients with AGE Reader, which is a validated method of SAF measurements. Previously, Yim et al. [35] using EcoSkin fluorescence video dermatoscope found increased autofluorescence in lesional psoriatic skin, significantly correlated with erythema in patients with psoriasis (*n* = 30) from South Korea. In this study, the autofluorescence of nonlesional skin was not increased. Authors did not collect data concerning comorbid conditions and BMI as well as ESR, HbA1c, and LDL levels in the study group. There is difficulty comparing the results because of different study designs and different methods used.

In the present study, we identified factors, such as systemic inflammation, prediabetes, and aging, which may influence enhanced AGE accumulation in individuals with psoriasis without any comorbidities. Increased AGE accumulation may accelerate the atherosclerosis and contribute to increased cardiovascular risk. Therefore, SAF may be considered as a useful, noninvasive method to identify patients with psoriasis at increased cardiovascular risk.

## Figures and Tables

**Table 1:** Clinical and demographic characteristics of the study group.

Characteristic	Patients with psoriasis (*n* = 70)	Controls (*n* = 59)
Mean age ± SD (range) years	49.33 ± 17.95	47.41 ± 14.11
Male *n* (%)/female *n* (%)	35 (50%)/35 (50%)	29 (49.15%)/30 (50.84%)
BMI mean ± SD	26.12 ± 3.39	25.22 ± 2.62
PASI mean ± SD	10.85 ± 6.57	
BSA (%) mean ± SD	23.28 ± 15.44	

**Table 2:** Characteristics of patients with chronic plaque psoriasis.

Characteristic	PsV (*n* = 70)	PsV type I (*n* = 49)	PsV type II (*n* = 21)	PsV with elevated CRP (*n* = 19)	PsV with increased ESR (*n* = 34)	PsV with borderline LDL (*n* = 16)	PsV with prediabetes (*n* = 21)	Controls (*n* = 59)
Age mean ± SD (years)	49.33 ± 17.95	42.92 ± 16.13	63.55 ± 11.93	54.79 ± 18.96	54.43 ± 16.38	52.50 ± 13.21	57.95 ± 11.55	47.05 ± 13.93
Male *n* (%)/ female *n* (%)	35 (50%) 35 (50%)	24 (48.97%) 25 (51.03%)	11 (52.39%) 10 (47.61%)	10 (52.63%) 9 (47.37%)	16 (47.05%) 18 (52.95%)	7 (43.75%) 9 (56.25%)	10 (47.61%) 11 (52.39%)	29 (49.1%) 30 (50.8%)
PASI mean ± SD	10.85 ± 7.07	11.02 ± 6.43	10.44 ± 7.021	11.88 ± 5.98	10.92 ± 5.832	10.02 ± 4.457	10.46 ± 4.4	
BSA mean ± SD (%)	23.39 ± 15.94	23.80 ± 13.15	22.40 ± 16.44	25.25 ± 16.06	23.38 ± 14.33	17.98 ± 13.09	18.44 ± 10.52	
BMI mean ± SD	26.12 ± 3.39	25.88 ± 3.66	26.68 ± 2.656	27.89 ± 2.031	27.16 ± 3.06	26.77 ± 2.662	28.33 ± 1.57	
CRP mean ± SD (mg/dl)	5.96 ± 9.79	4.63 ± 5.25	9.086 ± 15.80	15.45 ± 15.52	9.04 ± 12.54	6.306 ± 8.143	5.473 ± 4.751	
ESR mean ± SD (mm)	16.83 ± 13.89	13.50 ± 9.39	24.35 ± 19.51	23.28 ± 18.57	26.13 ± 14.44	15.77 ± 9.339	22.76 ± 11.70	
LDL mean ± SD (mg/dl)	108.30 ± 28.85	106.0 ± 27.97	113.7 ± 30.82	114.9 ± 23.54	108.5 ± 29.24	147.4 ± 7.164	117.8 ± 26.65	
HbA1c mean ± SD (%)	5.38 ± 0.47	5.297 ± 0.446	5.579 ± 0.46	5.6 ± 0.59	5.49 ± 0.55	5.453 ± 0.151	5.94 ± 0.226	
SAF mean ± SD	1.89 ± 0.59	1.665 ± 0.391	2.431 ± 0.638	2.037 ± 0.446	2.059 ± 0.52	2.093 ± 0.59	2.213 ± 0.526	1.816 ± 0.38

PsV: patients with chronic plaque psoriasis.

**Table 3:** Characteristics of patients with psoriasis according to gender.

Characteristic	Patients with psoriasis (*n* = 70)	Patients with psoriasis—*male* (*n* = 35)	Patients with psoriasis—*female* (*n* = 35)
Age mean ± SD (years)	49.33 ± 17.95	47.82 ± 16.86	49.94 ± 18.61
Male *n* (%)/ female *n* (%)	35 (50%) 35 (50%)		
PASI mean ± SD	10.85 ± 7.07	9.7 ± 4.452	11.98 ± 8.069
BSA mean ± SD (%)	23.39 ± 15.94	21.50 ± 12.30	25.27 ± 18.03
BMI mean ± SD	26.12 ± 3.39	26.45 ± 3.06	25.78 ± 3.71
CRP mean ± SD (mg/dl)	5.96 ± 9.79	5.66 ± 9.114	6.26 ± 10.54
ESR mean ± SD (mm)	16.83 ± 13.89	16.76 ± 16.70	16.52 ± 10.76
LDL mean ± SD (mg/dl)	108.30 ± 28.85	111.7 ± 27.91	105.0 ± 29.78
HbA1c mean ± SD (%)	5.38 ± 0.47	5.36 ± 0.49	5.403 ± 0.447
SAF mean ± SD	1.89 ± 0.59	1.843 ± 0.59	1.93 ± 0.57

## Data Availability

The data used to support the findings of this study are available from the corresponding author upon request.
